# Acute extreme hypocapnia and diabetic coma in an elderly patient after surgery: A case report

**DOI:** 10.1177/2050313X241282761

**Published:** 2024-09-21

**Authors:** Thomas Strecker, Michaela Siegle, Frank Bremer

**Affiliations:** 1 Center of Cardiac Surgery, Friedrich-Alexander-University Erlangen-Nuremberg, Erlangen, Germany; 2 Department of Anesthesiology, Friedrich-Alexander-University Erlangen-Nuremberg, Erlangen, Germany

**Keywords:** Diabetes, hyperglycemia, hypocapnia, respiratory medicine, surgery

## Abstract

Hyperglycemia and diabetic ketoacidosis are serious and life-threatening emergencies in diabetes patients. Early recognition of the symptoms of these disorders and their management are essential. Therapy is adequate rehydration, insulin treatment, electrolyte replacement, and handling of the underlying causative disease. Herein, we present an 83-year-old male with an extremely altered blood gas analysis after a surgical procedure of his left hand due to a phlegmon and describe the successful treatment through intensive care.

## Introduction

Hypocapnia means a reduced partial pressure of carbon dioxide (CO_2_) in the arterial blood. It occurs either as part of hyperventilation due to increased exhalation of CO_2_ or in the case of respiratory compensation of metabolic acidosis. Hypocapnia can lead to cerebral vasoconstriction, which in turn can lead to hypoxia, mental confusion, and ventilation problems and hypocapnia is an independent predictor of in-hospital mortality for acute heart failure.^1,2^

The combination of hypocapnia, diabetic ketoacidosis, and hyperglycemia is serious acute metabolic complication in diabetes patients. Management of these symptoms requires admission to the intensive care unit (ICU) and treatment of the precipitating underlying events.

## Case presentation

The 83-year-old Caucasian male was admitted to our university hospital due to a phlegmon of the left hand. Medical history contained arterial hypertension, type II diabetes, chronic obstructive pulmonary disease, and a transient ischemic attack 7 months ago. Since a right thalamic infarction 6 months ago, he has suffered from mild left hemiparesis. Furthermore, he suffers from a chronic wound-healing disorder after borehole trepanation with the evacuation of a traumatic left frontoparietal subdural hematoma 5 months ago.

Routine medication prior to hospital administration to control type II diabetes and hypertension was as follows: acetylsalicylic acid 100 mg/day, metformin 1000 mg/day, sitagliptin 100 mg/day, empagliflozin 10 mg/day, candesartan 4 mg/day and simvastatin 20 mg/day. Further information due to metabolic control was lacking, but previous episodes of decompensation were not reported by the relatives.

On the day of admission to our ICU from the normal ward, the patient had minor surgery due to a phlegmon in his left hand. Postoperatively, the patient had been experiencing progressively reduced consciousness and breathing difficulties, both of which had worsened extremely in the last couple of hours. Blood sugar level of 540 mg/dL (standard value 70–100 mg/dL) was detected and consequently, the patient was transferred to the ICU.

**Figure 1.**
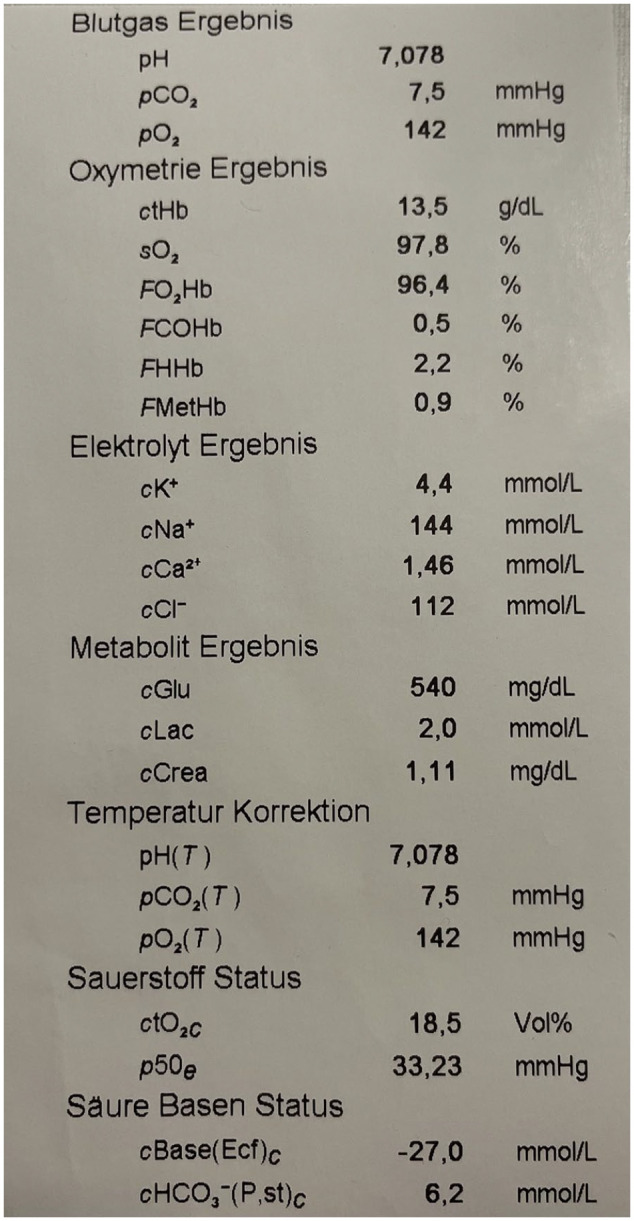
Blood gas analysis at admission. The patient presented in a comatose state with Kussmaul breathing.

The first blood gas analysis in the ICU revealed extreme hypocapnia with partial arterial pressure PaCO_2_ values of 7.5 mmHg (standard value 35–45 mg/dL) and a pH value of around 7.027 (standard value 7.35–7.45), respectively (Figure 1). The anion gap was significantly increased at 27, in accordance with the suspected diagnosis of severe metabolic ketoacidosis, serum lactate levels were almost within the normal range. The patient showed the typical signs of Kussmaul’s breathing with deep and frequent breaths, which in many cases precede a diabetic coma.

After ruling out cardiac failure with normal findings for transthoracic echocardiography, electrocardiogram, and myocardial infarction parameters (troponin I), volume substitution using ringer solution and norepinephrine was used for hemodynamic support. Blood sugar was controlled by continuous infusion of insulin aiming for levels below 200 mg/dL. Acidosis was reverted cautiously by sodium bicarbonate. Subsequently, the patient increasingly cleared up and could be contacted as before. Mechanical ventilation was not necessary, as oxygen saturation levels were good and there were no signs of respiratory muscle exhaustion.

Initially, higher catecholamine requirements decreased significantly with pronounced volume substitution. Due to the acidosis compensation, there was a slight hypernatremia of 154 mmol/L (standard value 135–145 mmol/L). Further clinical and microbiologic examinations revealed a severely elevated white blood cell (leukocyte) count 47.2 × 10^9^/L (standard value 4.1 × 10^9^/L), a high elevated C-reactive protein 933 nmol/L (standard value <47 nmol/L), a normal procalcitonin (PCT) 0.48 µg/L (standard value <0.5 µg/L) and evidence of staphylococcus aureus in the urinary tract. Therefore, antimicrobial therapy was escalated to meropenem and Fosfomycin in order to treat septic urinary tract infections. In the further course, we de-escalated the therapy to meropenem according to the antibiogram.

Due to the above-described subdural hematoma and the subsequent trepanation, the patient had to undergo a skull computed tomography scan to rule out cerebral pathologies as an explanation for the actual disease. No inflammatory complications such as an intracerebral abscess, intracerebral hemorrhages, or other processes referring to the actual disease could be found.

Within 48 h after admission to the ICU blood gas analysis, hemodynamics as well as metabolic and mental state normalized (relevant data see Table 1). Catecholamines could be stopped, and inflammatory parameters had decreased almost into the normal range: temperature 36.4°C, leukocytes of 9.4 × 10^9^/L, PCT 0.35 µg/L. Four days after admission to the ICU the patient could be transferred to the normal ward again. Finally, after further recovery, the patient was discharged to rehabilitation of two and a half weeks after admission to our hospital.

**Table 1.** Blood laboratory values during the stay in the ICU.

Hours after admission	0	1	2	3	5	7	8	9	12	17	19	24	29	33	37	40	44	48	52	55	57	60	63	65
pH	7.027	7.078	7.065	7.23	7.37	7.423	7.42	7.38	7.4	7.4	7.41	7.368	7.416	7.433	7.38	7.37	7.44	7.45	7.42	7.42	7.47	7.45	7.45	7.45
PaCO_2_ (mmHg)	10.3	7.5	11	13.5	16.1	17.7	19.6	20.8	20.6	24.7	23.7	28.1	27.6	26.9	23.6	19.9	25.3	30.1	30.2	29.4	32.5	33.2	33.2	36.2
HCO_3_^−^	5.9	6.2	6.7	9.6	13.6	15.8	16.6	15.7	16.3	18.2	17.9	18.1	19.8	20.3	16.8	14.8	19.6	22.7	21	20.6	24.9	24.4	26.7	27.8
BE	−26.7	−27	−25.8	−21.2	−15.5	−12.4	−11.2	−12.2	−11.3	−8.6	−9.1	−8.5	−6.3	−5.8	−10.4	−13.2	−6.6	−2.6	−4.6	−5.1	0.1	−0.4	2.2	3.5
Potassium (mmol/L)	4.3	4.4	4.3	4.3	3.4	3.7	4	4.2	3.9	4.5	4.3	3.8	4.2	4.2	4.3	4.3	4.1	4	4	4.1	4.5	3.9	3.8	4.3
Sodium (mmol/L)	142	144	144	147	152	154	154	151	152	152	153	155	156	154	155	153	153	153	152	153	152	153	154	153
Calcium (mmol/L)	1.42	1.46	1.41	1.33	1.21	1.31	1.31	1.29	1.32	1.28	1.3	1.26	1.25	1.23	1.28	1.3	1.29	1.27	1.3	1.3	1.3	1.29	1.29	1.26
Chlorid (mmol/L)	110	112	113	114	113	114	115	118	118	121	123	120	120	121	120	120	121	124	123	122	119	117	118	118
Glucose (mg/dL)	409	540	530	482	342	274	243	215	256	243	220	228	198	172	189	236	168	123	163	206	199	169	140	136
Lactat (mg/dL)	1.9	2	2.1	2.6	1.8	2.1	1.6	1.6	1.1	1	0.9	0.8	0.7	0.8	1.3	0.7	0.8	1.1	0.7	0.5	0.6	0.7	0.6	0.7
Creatinin (mg/dL)	0.74	1.11	1.1	0.98	0.97	0.96	0.99	0.94	0.91	0.82	0.81	0.8	0.7	0.66	0.7	0.7	0.7	0.7	0.6	0.7	0.65	0.6	0.6	0.6
Norepinehrin (mg/h)	0.4	0.4	0.6	0.6	1	1.1	1	1.2	1.3	1	1	0.9	0.9	0.8	0.4	0.1	0							
Insulin (iE/h)	2	2	2	2	2	2	2	1.5	1.5	1.5	1	1	0.5	0.5	0.5									
Sodium bicarbonate (mmol)			100					50																
Anion gap (mmol/L)	30.4	30.2	28.6	27.7	28.8	27.9	26.4	21.5	21.6	17.3	16.4	20.7	20.4	16.9	22.5	22.5	16.5	10.3	12	14.5	12.6	15.5	13.1	11.5

ICU: intensive care unit.

BE: base excess

## Discussion

In the following section, we want to discuss the presented case report especially addressing severe acidosis, urosepsis, and medication history.

Unfortunately, we didn’t measure ketone bodies but lactate concentration was quite normal. We presume that high values of an augmented anion gap (Table 1) were caused by ketoacidosis. In our case, the patient was taking metformin, empagliflozin, and sitagliptin to control type 2 diabetes. Although rarely occurring ketoacidosis is a well-known side effect of metformin as well as empagliflozin therapy.^3,4^

Pharmacological effects of SGLT2 inhibitors are mainly conveyed by inhibition of tubular reabsorption of glucose in the kidneys. As a possible result increased incidence of urinary tract infections are discussed in the literature.^5,6^ In this case report our patient presented staphylococcus aureus urosepsis as one of the main clinical features.

SGLT2 inhibitors may also trigger osmotic diuresis and hypovolemia.^7,8^ The latter side effect may be worsened by capillary leak syndrome and edema formation in the presence of urosepsis, as seen in our case.

Despite the severity of this medical situation with extreme hypocapnia, hyperglycemia, and dehydration, there are only few scientific medical studies for the best treatment strategies.^9,10^ To our knowledge, this is the first case report of hypocapnia with PCO_2_ values of 7.5 mmHg and sugar levels of 540 mg/dL in an adult patient with survival. The therapy of this condition is directed by eliminating hyperventilation, adjusting the blood glucose, balancing the volume deficit and the electrolyte parameters as well as the treatment of the underlying illness that triggered metabolic decompensation.^11,12^

## Conclusion

Metabolic acidosis is a serious metabolic disbalance due to insulin deficiency that requires immediate intensive care medical treatment. Hyperventilation as an attempt to compensate for this acidosis leads to a reduction in the partial arterial pressure. The keystone of treatment for this life-threatening disease is the intravenous infusion of fluids, insulin, and electrolytes.
